# Interventions on informal healthcare providers to improve the delivery of healthcare services in low-and middle-income countries: a systematic review

**DOI:** 10.3389/fpubh.2024.1456868

**Published:** 2024-10-01

**Authors:** Saibal Das, Shweta Khare, Jaran Eriksen, Vishal Diwan, Cecilia Stålsby Lundborg, Kristina Skender

**Affiliations:** ^1^Department of Global Public Health, Karolinska Institutet, Stockholm, Sweden; ^2^Indian Council of Medical Research - Centre for Ageing and Mental Health, Kolkata, India; ^3^Department of Public Health Sciences and Environment, RD Gardi Medical College, Ujjain, India; ^4^Department of Clinical Science and Education, Södersjukhuset, Karolinska Institutet, Stockholm, Sweden; ^5^Indian Council of Medical Research - National Institute for Research in Environmental Health, Bhopal, India

**Keywords:** healthcare services, informal healthcare providers (IHCPs), intervention, low-and middle-income countries Normal, left

## Abstract

**Objective:**

Informal healthcare providers (IHCPs) play a big role in health systems in low-and middle-income countries (LMICs) and are often the first point of contact for healthcare in rural and underserved areas where formal healthcare infrastructure is insufficient or absent. This study was performed to systematically review the literature on interventions targeting IHCPs in improving the delivery of healthcare services in LMICs.

**Methods:**

PubMed, Embase, and Cochrane CENTRAL databases were searched for studies that assessed any type of intervention among IHCPs to improve the delivery of healthcare services in any LMIC. Outcomes included changes in knowledge, attitude, and reported practice of appropriate case diagnosis and management; improved referral services; effective contraceptive use; and medication appropriateness (PROSPERO ID: CRD42024521739).

**Results:**

A total of 7,255 studies were screened and 38 were included. Most of the studies were conducted in Africa and Asia. The IHCPs who were trained included medicine sellers, community health workers/traditional healers, and traditional birth attendants. The main intervention used was educational programs in the form of training. The other interventions were health services, policy and guidelines, and community-based interventions. Most of the interventions were multi-faceted. The disease/service areas targeted were mainly maternal and child health, sexually transmitted diseases, common infectious diseases, medicine use/dispensing practices, and contraception. The outcomes that showed improvements were knowledge, attitude, and reported practice; diagnosis and case management; improved referral services; contraceptive uses; and medication appropriateness. Around one-fourth of the studies reported negative results. The certainty of evidence generated (GRADE criteria) was very low.

**Conclusion:**

Some multifaceted interventions coupled with training showed improvements in the delivery of healthcare services by IHCPs. However, the improvements were inconsistent. Hence, it is unclear to identify any context-specific optimum intervention to improve the delivery of healthcare services by IHCPs.

## 1 Introduction

Informal healthcare providers (IHCPs) play a big role in providing healthcare services in low-and middle-income countries (LMICs), especially in places like India, Bangladesh, and various parts of Africa due to their accessibility, affordability, and familiarity within the community (1–3). IHCPs practice allopathic and non-allopathic medicine with minimal or no formal training and constitute a significant portion of the private healthcare sector (1–3). IHCPs are broadly categorized based on the nature of their practice. This encompasses retail medicine sellers, traditional healers, faith healers, traditional birth attendants, untrained allopathic providers, and traditional medicine practitioners (1, 2). Additionally, IHCPs include individuals trained in one field but practicing in another, such as nurses offering medical consultations as doctors within their communities (1).

IHCPs are often the first point of contact for healthcare in rural and underserved areas where formal healthcare infrastructure is insufficient or absent. The gap in healthcare systems in LMICs is primarily due to inadequate infrastructure, a shortage of formally trained healthcare professionals, and logistical challenges in reaching remote areas. Limited resources and funding constraints often mean that formal healthcare facilities are sparse, poorly equipped, or concentrated in urban centers, leaving rural and impoverished communities underserved. IHCPs, despite their lack of formal training, attempt to fill this gap by offering various health services, often tailored to the specific needs and cultural contexts of their communities (1, 2). People seek out the services of IHCPs for a range of interventions, including preventive, curative, and restorative care.

Despite their significant role, IHCPs are often overlooked in the healthcare system, which particularly affects the poor who rely on them. Unlike formal private actors, IHCPs lack recognition within a country’s regulatory and legal framework, despite being sought after by the clients they serve. Payment is usually received directly from patients, without documentation, rather than from institutions (2). They may also belong to professional associations that lack certification or regulatory authority (2, 4). Many IHCPs may be hesitant to formalize their practice, especially if government penalties exist for those who advertise themselves illegally as legitimately trained professionals (2, 4).

Despite the renewed emphasis on primary healthcare, surprisingly few studies have explored the potential of the informal sector to extend healthcare to communities (5). Interventions recognizing IHCPs’ contribution to healthcare provision could help legitimize their profession and identify opportunities to expand essential healthcare in LMICs in alignment with public health goals of quality and affordability. Training IHCPs could potentially enhance healthcare in these communities, but implementing such an approach necessitates identifying these providers, their practice locations, and the specific training they require. Currently, there is a lack of summarized data about the types of interventions and their effectiveness in improving the quality of healthcare delivery provided by IHCPs. Conducting a systematic review of interventions targeting IHCPs is essential to consolidate existing knowledge and evaluate the effectiveness of these interventions in enhancing healthcare delivery in LMICs. Given the significant role IHCPs play in providing accessible healthcare services, especially in rural and underserved areas, a comprehensive review was aimed at identifying successful interventions.

## 2 Methods

### 2.1 Search strategy and information sources

The researchers conducted a literature search in the PubMed, Embase, and Cochrane CENTRAL electronic databases for original interventional studies published in English from LMICs between 1990 and June 2024. The search was last conducted on 30 June 2024. Systematic reviews, qualitative studies, editorials, commentaries, conference proceedings, and case series/reports were not included. Various search terms were utilized, as outlined in Supplementary Table S1. These search terms were adapted for different bibliographic databases, incorporating database-specific filters. Two independent authors identified relevant studies based on their titles and abstracts using the search strategy. They then obtained the abstracts and, if necessary, the full texts of the studies to evaluate their suitability for inclusion. The discrepancies were resolved by discussion with the arbiter.

### 2.2 Eligibility criteria and study selection

**Figure 1:**
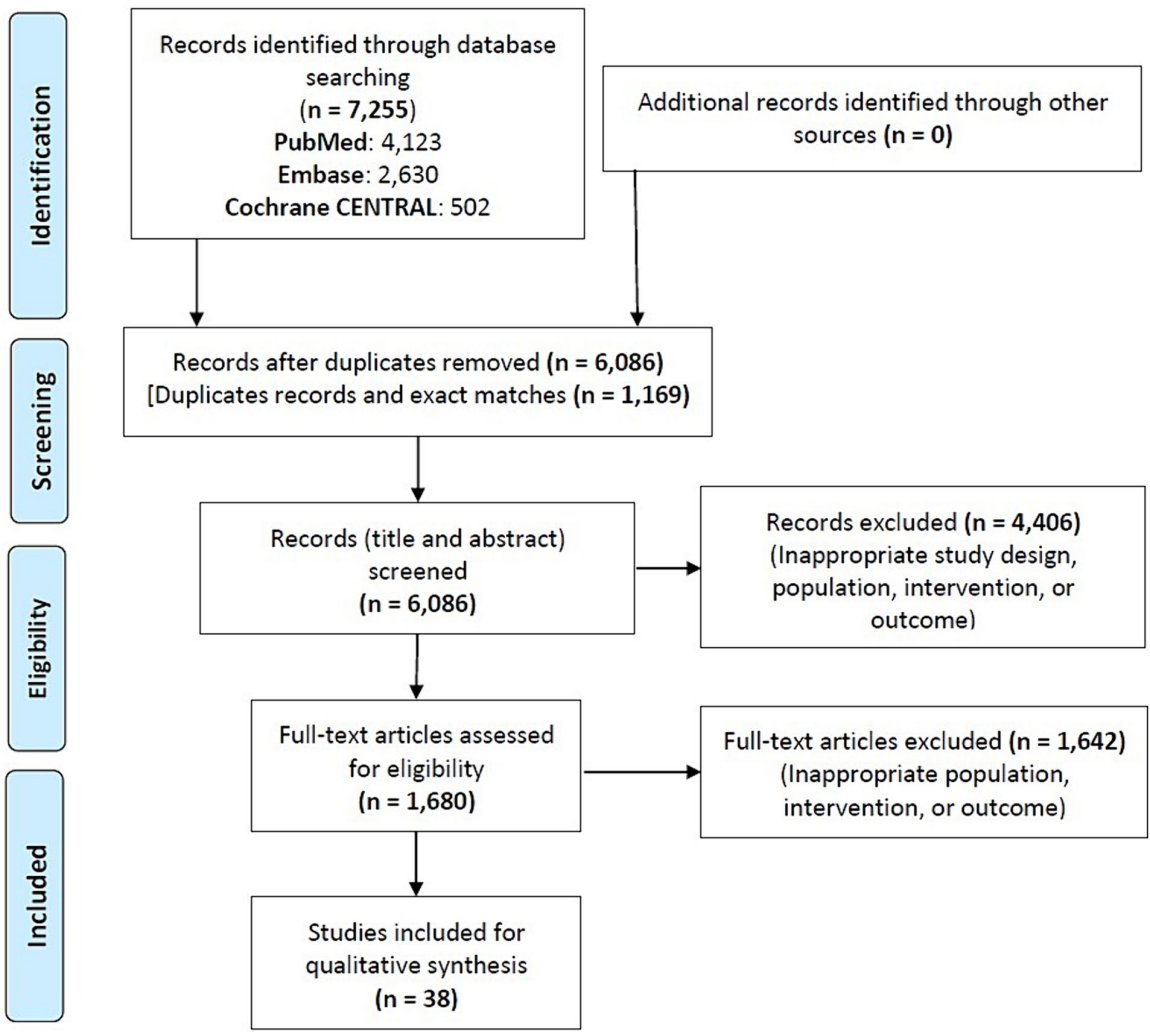
Study flowchart.

Since the informality of care varies depending on the context, rigid criteria cannot universally define IHCPs. Therefore, a set of operational criteria was established to define IHCPs operating in LMICs: individuals lacking formal training in an institution recognized and affiliated by the regulatory authority/council of a particular country; those without registration, regulation, or oversight by any institution or governing body for their practice; and those working in a private setting whose scope of work is not recognized by the regulatory authority/council of that particular country (2). Community health workers trained and recognized by non-governmental organizations and governments, such as the Accredited Social Health Activist women workforce in India, who act as the first point of contact for any health-related issue, were excluded. Original interventional studies assessing any type of intervention (educational, behavioral, social, etc.) among IHCPs to enhance the delivery of healthcare services in any setting in any LMIC were included. The comparator was no intervention or any specific active intervention. Outcomes included changes in knowledge, attitude, and reported practice of appropriate case diagnosis and management; improved referral services; effective contraceptive use; and medication appropriateness. Specifically, studies were not included if they did not meet our predefined selection criteria, such as studies that were not quasi-experimental or randomized controlled trials (inappropriate study design) or studies that did not focus on IHCPs in LMICs based on our operational criteria (inappropriate population) (Figure 1).

### 2.3 Data items and analysis

A standardized, pre-formatted form was used to extract data from the eligible studies. The extracted information encompassed various aspects, including the study setting, study population (type of IHCPs), participant demographics and characteristics, details of interventions and comparators, and outcomes. The data was managed using Microsoft Excel. Descriptive statistics were used to summarize the results. Additionally, The World Health Organization (WHO) Health Intervention Classification Framework categories were used to classify the interventions as follows (62):

(1) Education and training: activities aimed at improving knowledge and skills.
(2) Health services: direct healthcare services provided to individuals.
(3) Policy and guidelines: implementation of policies or guidelines to improve health outcomes.
(4) Community-based interventions: activities that engage community members in health promotion and disease prevention.

### 2.4 Risk of bias assessment

For the risk of bias analysis, the ROBINS-I tool (6) was used for quasi-experimental studies and the Cochrane risk of bias tool 2 (59) was used for randomized controlled trials by an independent author. The ROBINS-I tool includes the assessment of the following biases: confounding, selection of participants in the study, classification of interventions, deviations from intended interventions, missing data, measurement of outcomes, selection of the reported result, and overall. The Cochrane risk of bias tool 2 includes the assessment of the following biases: randomization process, deviations from intended interventions, missing outcome data, measurement of the outcome, selection of the reported result, and overall. Attrition rates, including dropouts, loss to follow-up, and withdrawals, were also examined. Issues of missing data and imputation methods were critically evaluated.

### 2.5 Certainty assessment

The GRADE (Grading of Recommendations Assessment, Development and Evaluation) approach was used to assess the certainty of the generated evidence (7, 57).

### 2.6 Study protocol

This systematic review complies with the Preferred Reporting Items for Systematic Reviews and Meta-Analyses (PRISMA) guidelines. The study protocol was registered in the International Prospective Register of Systematic Reviews (PROSPERO ID: CRD42024521739).

## 3 Results

### 3.1 Study selection, characteristics, and results of quality assessment

A total of 7,255 studies were screened and finally, 38 were included (Figure 1). The studies were published between 1992 and 2021. The results of the risk of bias analysis are itemized in (Supplementary Tables S2, S3). Eight quasi-experimental studies had high risk of bias, while the randomized controlled trials had a moderate or low risk of bias. The summary of the study characteristics is enumerated in Table 1. The quasi-experimental study design was used in most of the cases (68%). Most of the studies were conducted in Africa (50%) and Asia (40%). The sample size ranged from 17 to 1,133.

**Table 1:** Summary of the study characteristics (*n* = 38).

Author, year	Country	Study design	Population	Intervention	Comparator	Outcomes	Key findings	*n* in the intervention arm	*n* in the control arm
Oshiname, 1992 (30)	Nigeria	Quasi-experimental	Medicine sellers	Training (8 weekly 2-h sessions)	No training	Recognition and treatment for malaria, diarrhea, guinea worm, STDs, respiratory infections, and malnutrition, plus sessions on reading prescriptions and medication counseling	Improved knowledge of treatment for malaria, diarrhea, guinea worm, STDs, respiratory infections, and malnutrition, plus medication counseling post-training	37	–
Podhipak, 1993 (31)	Thailand	Quasi-experimental	Medicine sellers	Intervention program	No intervention	Changes in the prescription of ORS, antibiotics, and antidiarrheal medicines	Post-intervention, there was no change in ORS, antibiotics, and antidiarrheal medicines prescribed to treat watery diarrhea by medicine sellers, while in dysentery, the prescribing of ORS improved. For medicine sellers, ORS usage increased for treating watery diarrhea, but not for dysentery. There was a slightly significant change in behavior concerning the use of antibiotics	–	–
Kambo, 1994 (32)	India	Quasi-experimental	Traditional medical practitioners	Training for 2 years	No training	Delivery of contraceptive services	Training led to an increased knowledge of contraceptive use	–	–
Lynch, 1994 (33)	Uganda	Quasi-experimental	Traditional birth attendants	Training	No training	Effective performance and utilization of TBAs	Trained TBAs attended 3 times the number of deliveries; however, there was no difference in the knowledge, attitude, reported practice, and performance of TBAs following training	40	40
Alisjahbana 1995 (10)	Indonesia	Quasi-experimental	Informal care providers	Training at all levels of the health care system and establishment of birthing homes in villages	No training	Referral, transportation, communication, and appropriate case management	Post training, there were improvements in the antenatal care coverage, reduction in post-partum complications, better case referrals, and mixed results in terms of perinatal death	–	–
Kumar, 1995 (34)	India	Quasi-experimental	Traditional birth attendants	Training (1 day per month)	No training	Case management of birth asphyxia in-home deliveries and perinatal mortality	There were improved resuscitation outcomes and a reduction in perinatal mortality	100	–
Matthews, 1995 (35)	Nigeria	Quasi-experimental	Traditional birth attendants	Training	No training	Improvement of care of mothers and babies	Training improved the identification of high-risk pregnancies and improved the care of mothers and babies	–	–
Miller, 1995 (36)	Pakistan	Quasi-experimental	Traditional birth attendants	Training	No training	Perinatal outcomes; knowledge, attitude, and reported practice on breastfeeding; maternal nutrition; immunization and hygiene	Training improved knowledge and skills about breastfeeding, maternal nutrition, immunization, and hygiene. Training also reduced complications and deaths associated with deliveries	–	–
Pick, 1996 (37)	Mexico	Quasi-experimental	Medicine sellers	Intensive 8-h training course reinforced by appropriate instructional and promotional materials	No training	Knowledge retention about AIDS/HIV, willingness to convey accurate knowledge, and increase in condom sales	Although training increased short-term knowledge about HIV transmission and prevention, it was less successful in achieving long-term information retention, transfer of knowledge to clients, or influencing condom sales	166	–
Nations, 1997 (38)	Brazil	Quasi-experimental	Afro-Brazilian Umbanda healers	Multidisciplinary training	No training	knowledge, attitude, and reported practice regarding the prevention of AIDS	Training led to a significant increase in AIDS awareness, knowledge about risky HIV behavior, information about correct condom use, alternative ritual blood practices, and a reduction in prejudicial attitudes related to HIV transmission	126	100
Somsé, 1998 (61)	Central African Republic	Quasi-experimental	Traditional healers	Training (17–36 h)	No training	Knowledge and attitude towards treatment of AIDS and STDs	Improvement in knowledge and/or attitudes was observed toward the treatment of AIDS and STDs except for the prevention of HIV transmission	96	–
Marsh, 1999 (39)	Kenya	Quasi-experimental	Medicine sellers	Training (each for 3 days)	No training	Changes in the sales of antimalarial or antipyretic medicines, the total number of chloroquine tablets purchased, and the reported use of shop-bought medicines for children	Training improved the sales and use of antimalarial medicines for fever in children and increased the appropriate use of chloroquine	46	–
Schaider, 1999 (40)	Angola	Quasi-experimental	Traditional birth attendants	Training (38 h) on prenatal, delivery, and postnatal care	No training	Maternal mortality rate	Maternal mortality rate was reduced post-training	1,133	–
Adu-Sarkodie, 2000 (41)	Ghana	RCT	Medicine sellers	Training	No training	Syndromic management of STIs	Training led to improvements in the treatment of urethral discharge	50	50
Smith, 2000 (42)	Ghana	Quasi-experimental	Traditional birth attendants	Training	No training	Maternal and Perinatal outcomes	The beneficial impact of training was not compelling	–	–
Singhal, 2001 (8)	Philippines	RCT	Traditional birth attendants	Lecture-style educational program	Interactive problem-based educational program	knowledge, attitude, and reported practice in maternal care, birth, and neonatal care	There was a significant improvement in knowledge, attitude, and certain practices; however, a need for further education and reinforcement was reported	31	20
Bailey, 2002 (43)	Guatemala	Quasi-experimental	Traditional birth attendants	Training intervention	No training	Detection of obstetric complications, referral of patients with complications, and, utilization of essential obstetric care services	Training traditional birth attendants had a positive effect on the rate, detection, and referral of postpartum complications; however, the evidence was less convincing for an overall increase in the detection of complications, referral to the formal health care system, and the utilization of essential obstetric services	–	–
Chalker, 2002 (44)	Vietnam	RCT	Medicine sellers	Multi-component intervention on knowledge and reported practice regarding ARI, STD, and antibiotic/steroid requests (3 interventions over 17 months)	No training	Knowledge and reported practice of STD, ARI, and non-prescription requests for antibiotics and steroids	Training improved knowledge and reported practice of ARI and STD treatment and sale of antibiotics	25	25
Ratanajamit, 2002 (45)	Thailand	Quasi-experimental	Medicine sellers	Educational program	No education	Knowledge of and reported practice in dispensing emergency contraception	Education led to a significant improvement in the choice of medicine, advice provided, and knowledge of the time limit for initiating emergency contraception; however, proper history taking on the time of intercourse and menstrual cycle did not improve	60	60
Garcia, 2003 (18)	Peru	RCT	Medicine sellers	Training (interactive seminars on recognition and management of four STD syndromes, monthly pharmacy visits by “prevention salespersons,” and workshops)	No training	Recognition and management of STDs	Training led to significantly better recognition and management (appropriate antimicrobial regimens) for STDs, significantly more frequent recommendations for the use of condoms, and treatment of partners at pharmacies	220	220
Kaona, 2003 (13)	Zambia	Quasi-experimental	Village health motivators and sellers	Deployment of anti-malarial medicine sellers and village health motivators	No intervention	Identify malaria and correctly use chloroquine	Post-intervention, mothers and other caretakers were more likely to identify simple and severe malaria and there was a 60% increase in correct chloroquine	345	230
Poudyal, 2003 (46)	Nepal	RCT	Traditional healers	Western medical training model to upgrade basic knowledge about common illnesses including HIV/AIDS and to improve referral practices	No training	Knowledge of common diseases, referral practice	Training improved knowledge of allopathic medicine and referral practices	48	30
Tavrow, 2003 (47)	Kenya	Quasi-experimental	Medicine sellers	Outreach education (shopkeeper job aid, client awareness aid, orientation of medicine wholesale owners, training and equipping of mobile medicine sellers, medicine wholesale counter attendants, and monitoring)	No intervention	Private sector’s compliance with malaria guidelines	The intervention had a significant impact on medicine stocking patterns, malaria knowledge, and prescribing practices of shops/kiosks, but not consistently on other types of outlets	101	151
Tumwikirize, 2004 (48)	Uganda	Quasi-experimental	Medicine counter attendants	Face-to-face educational intervention	No intervention	Dispensing behavior for mild and severe ARI in children at private pharmacies and medicine shops	Despite training, the assessment of the child’s condition remained inadequate, inappropriate dispensing practices were frequent, antibiotic prescribing was very common, and barely any advice or instruction was given with dispensed medicines	191	–
Chalker, 2005 (49)	Vietnam, Thailand	RCT	Medicine sellers	Multi-faceted intervention (three 3-month interventions over 4 months)	No training	Dispensing practices (steroids, antibiotics) and providing advice	The intervention resulted in a significant reduction in the dispensing of illegal steroids and low-dose antibiotics	73	73
Jokhio, 2005 (60)	Pakistan	cRCT	Traditional birth attendants	Training (training for 3 days on identification of danger signs in pregnancy)	No training	Perinatal and maternal mortality	Perinatal and maternal mortality were reduced post-training	10,114	9,443
Peltzer, 2006 (50)	South Africa	Quasi-experimental	Traditional healers	Training for 3.5 days in HIV/AIDS, STI, and TB prevention	No training	HIV knowledge and HIV and STI management strategies	Intervention effects were significant for knowledge and management strategies of HIV and STIs, including conducting risk behavior assessments and counseling, condom distribution, community education, and record keeping; however, the rate of referral was not improved	160	73
Hamid Salim, 2006 (58)	Bangladesh	Quasi-experimental	Village doctors	Training (one-day orientation and training course on TB)	No training	Referral and treatment of TB	Training resulted in improved quality of TB treatment	12,525	–
Tawfik, 2006 (51)	Uganda	Quasi-experimental	Formal and informal private practitioners	Negotiation sessions	No training	Management of childhood diarrhea, acute respiratory infection, and malaria	Post-intervention the quality of case management was generally better, although certain practices appeared resistant to change	73	–
Mbonye, 2007 (11)	Uganda	Quasi-experimental	Traditional birth attendants, medicine sellers, community reproductive health workers, and adolescent peer mobilizers	A novel community-based delivery system to deliver the preventive treatment of malaria in pregnancy	No intervention	Community delivery of intermittent preventive treatment of malaria in pregnancy	The community-based system was effective in delivering preventive treatment of malaria in pregnancy, the treatment adherence improved, the antenatal coverage improved, the proportion of women seeking care for malaria at health units increased, and the use of insecticide-treated nets increased	2,081	1,055
Nsimba, 2007 (12)	Tanzania	Quasi-experimental	Medicine sellers	Educational intervention approach	No intervention	Practices, compliance, and performance in using the national treatment guidelines for malaria and other common childhood illnesses (diarrhea and ARI)	The intervention significantly improved the knowledge for prescribing and dispensing of medicines for common childhood illnesses, including dispensing first-line anti-malarial medicines	20	20
Onwujekwe, 2007 (52)	Kenya	Quasi-experimental	Community health workers	Training	No training	Near and appropriate treatment of malaria	Post-training, community health workers provided malaria treatment services at low cost, and their market share of malaria treatment in the villages increased	–	–
Shah, 2007 (53)	Pakistan	RCT	Non-formal care providers	Training in syndromic management	No training	Quality of STD services	Training had a positive impact on the quality of STD case management service	–	–
Abuya, 2009 (54)	Kenya	cRCT	Medicine sellers	Training (workshop on selling antimalarials and public information campaigns on the use of over-the-counter antimalarials)	No training	Malaria treatment improvement	Training led to improved knowledge, attitude, and reported practice on the sale of antimalarials	74	67
(14)	India	RCT	Informal providers	Multitopic training program (72 sessions of training over 9 months)	No training	Adherence to condition-specific checklists, correct case management, and the use of unnecessary medicines and antibiotics	Training increased correct case management but did not affect the use of unnecessary medicines and antibiotics	388	396
Talukder, 2017 (9)	Bangladesh	cRCT	TBAs or community volunteers	Training for 5 days and post-training supervision (intervention 1)	Training for 5 days (intervention 2) and no training (control)	Early breastfeeding practices	Increased proportion of early initiation of breastfeeding and avoidance of pre-lacteal feeds post-training and supervision	321	400 and 461
Sima, 2019 (55)	Ethiopia	Quasi-experimental	Traditional healers	Training	No training	Detection and referral of active TB cases	Training led to an improvement in the detection of undiagnosed active TB cases in the community	22	–
Sundararajan, 2021 (56)	Uganda	RCT	Traditional healers	One-day educational training on HIV	No training	Delivery of point-of-care HIV tests	Delivery of point-of-care HIV tests by traditional healers increased significantly by more than 4 times following training	9	8

AIDS, acquired immunodeficiency syndrome; ARI, acute respiratory infection; cRCT, cluster randomized controlled trial; HIV, human immunodeficiency virus; ORS, oral rehydration solution; RCT, randomized controlled trial; STD, sexually transmitted disease; STIs, sexually transmitted infections.

### 3.2 Synthesis of study results

The main intervention used was educational programs in the form of training. These interventions included regular interactive educational training programs, both short-term (up to 3 months) and long-term (9 months to 2 years), aimed at improving knowledge in specified areas. Additionally, outreach education programs and interactive seminars were employed to enhance knowledge and management practices. The IHCPs who were trained included medicine sellers (34%), community health workers/traditional healers (34%), and traditional birth attendants (29%). The common disease/service areas that were targeted were maternal and child health (39%), sexually transmitted diseases/infections (STDs/STIs) (24%), and malaria (16%) (Table 2). We identified various types of interventions categorized based on their intended impact on provider behavior. Many interventions were implemented in combination with related strategies. Approximately 68% of all studies employed multiple intervention strategies, with nearly all studies measuring more than one outcome. The common strategy was management improvement, including training, provision of supplies, job aids, and financial incentives (market-based approach). However, the interventions were inconsistent and heterogeneous across studies. The comparator was no intervention in all studies except two (8, 9), where specific active interventions were used as a comparator.

**Table 2:** Summary of the findings (*n* = 38).

Parameter		No. of studies (%)
Study design	Quasi-experimental studies	26 (68.4)
	Randomized controlled trials	9 (23.7)
	Cluster randomized controlled trials	3 (7.9)
Continent	Africa	19 (50)
	Asia	15 (39.5)
	South America	4 (10.5)
Type of informal healthcare provider	Medicine sellers	13 (34.2)
	Community health workers/traditional healers	13 (34.2)
	Traditional birth attendants	11 (28.9)
	Others (mixed)	1 (2.7)
Broad disease/service areas targeted	Maternal and child health	11 (28.9)
	STDs including HIV	9 (23.7)
	Malaria	6 (15.8)
	Medicine use/dispensing practices	4 (10.4)
	Tuberculosis	2 (5.3)
	Childhood common diseases	2 (5.3)
	Contraception	2 (5.3)
	Mixed	2 (5.3)

HIV, human immunodeficiency virus; STD, sexually transmitted disease.

Twenty-eight studies reported changes in knowledge, attitude, and reported practice of appropriate case diagnosis and management after intervention for common conditions (respiratory infection, diarrhea, malaria, etc.), STDs/STIs, and perinatal care. Seven studies evaluated improvement in referral services post-intervention for perinatal care, STDs/STIs, and tuberculosis. Seven studies evaluated increased knowledge of contraceptive use and increased sales of condoms. Eight studies evaluated post-training improved medication appropriateness focusing on antibiotics for common diseases (respiratory infection, diarrhea, malaria, etc.). Among the studies, 26% reported positive outcomes with training alone, while 47% reported positive outcomes with training combined with other interventions. Most studies assessed provider performance as a basic measure of intervention effectiveness. Traditional healers showed the greatest improvement in provider knowledge. Post-training, improvements were observed in knowledge, attitude, and reported practice of appropriate case diagnosis and management; improved referral services; effective contraceptive use; and medication appropriateness. However, the improvements were inconsistent across studies. A high proportion (89%) of randomized controlled trials reported positive outcomes, while 26% of all studies reported one or more negative outcomes. The certainty of evidence generated (GRADE criteria) was; however, very low for all outcomes (Supplementary Tables S4–S8).

The interventions from the study primarily fall under the WHO Health Intervention Classification Framework categories of education and training, health services, policy and guidelines, and community-based interventions (Table 3; Supplementary Table S9) (World Health Organization, 2023). Education and training were the most common and reported in most of the studies, leading to improved knowledge and skills among healthcare providers, traditional healers, and community workers, often resulting in better health practices, increased treatment adherence, and reduced mortality rates. The service areas covered by education and training were common infectious diseases, STDs, malnutrition, and antenatal care. Health services interventions, such as the establishment of birthing homes in villages for perinatal care (10) or the establishment of a novel community-based delivery system for effective infectious disease management (11). Multi-component policy and guidelines intervention led to improved knowledge and reported practice of the treatment of acute respiratory infections and STDs and the sale of antibiotics (12). Lastly, community-based interventions demonstrated success in the deployment of anti-malarial medicine sellers and village health motivators for increased identification of malaria and improved correct chloroquine use (13).

**Table 3:** Summary of the interventions and outcomes based on the World Health Organization (WHO) Health Intervention Classification Framework categories.

Intervention classification	Type of interventions	Service area	Summary outcomes
Education and training	Regular training for short term (up to 3 months)	Common infectious diseases, STDs, malnutrition, and antenatal care	Improved knowledge of treatment for malaria, diarrhea, guinea worm, STDs, respiratory infections, and malnutrition; improved resuscitation outcomes and reduction in perinatal mortality
	Regular training for long term (9 months to 2 years)	Use of antibiotics and contraceptives	Increased knowledge of antibiotic use and contraceptive use
	Outreach education program for medicine sellers	Infectious disease (malaria)	Significant impact on medicine stocking patterns, malaria knowledge, and prescribing practices of shops/kiosks
	Interactive seminars on recognition and management of STDs and workshops	STDs	Better recognition and management of STDs, increased condom recommendations, and partner treatment
Health services	Establishment of birthing homes in villages	Antenatal care	Improvements in antenatal care coverage, reduction in post-partum complications, better case referrals, and mixed results in terms of perinatal death
	Establishment of a novel community-based delivery system	Infectious disease (malaria) and perinatal care	Effective community delivery of preventive treatment of malaria in pregnancy, improved treatment adherence, antenatal coverage, and care-seeking behavior
Policy and guidelines	Multi-component intervention on knowledge and reported practice regarding ARI, STDs, and antibiotic/steroid use	Common infectious diseases and STDs	Improved knowledge and reported practice of ARI and STDs treatment and sale of antibiotics
Community-based interventions	Deployment of anti-malarial medicine sellers and village health motivators	Infectious disease (malaria)	Increased identification of malaria and improved correct chloroquine use

ARI, acute respiratory STD, sexually transmitted disease.

Some interventions can fit into multiple categories, but to avoid overlap, they are placed under the most appropriate classification, emphasizing their primary focus as follows:

Education and training include all interventions with a primary focus on training and educational efforts.

Health services are specifically focused on the establishment of health services, such as birthing homes.

Policy and guidelines cover interventions that were primarily aimed at changing practices through multi-component or policy-driven efforts.

Community-based interventions include only those interventions focused on engaging the community through specific strategies, such as deploying health motivators.

## 4 Discussion

This systematic review aimed to summarize the evidence on various interventions in improving the delivery of healthcare services by IHCPs in LMICs. A total of 38 studies published between 1992 and 2021 were included. The majority of these were conducted in Africa and Asia, targeting IHCPs, including medicine sellers, community health workers, and traditional birth attendants. Interventions primarily focused on maternal and child health, STDs/STIs, and malaria employing educational programs in the form of training. Training included both short-term and long-term interactive educational sessions, along with outreach education programs and interactive seminars. Post-intervention improvements were observed in areas such as knowledge, diagnosis, referral services, contraceptive use, and medication appropriateness, although some studies reported negative outcomes. A context-specific optimum intervention strategy to improve healthcare delivery by IHCPs in LMICs was not identified overall.

Our results show that the interventions to improve the IHCPs’ delivery of healthcare services were heterogeneous and while the majority reported positive outcome(s), the improvements were inconsistent. Education and training were the most common interventions, leading to improved knowledge and skills of IHCPs, often resulting in improvement in health practices. However, when considering the training of IHCPs, greater attention is required to integrate it within a broader context of factors likely to promote better practices and reinforce the training. The effectiveness of training likely requires continuous efforts rather than one-time initiatives, and its retention should be evaluated at multiple points in time (14). As IHCPs operate within the private sector, their livelihood often depends on customer satisfaction and repeat business. As a result, interventions like external quality assessments, mandatory training programs, and the distribution of printed materials, which IHCPs believe do not affect their financial outcomes, have not been well customized (15, 16). Institutional innovations, like franchising, or locally tailored regulatory approaches, such as performance reviews, could create the necessary pressure for IHCPs to adhere to norms and deliver improved care (17). Training played a supplementary, yet potentially crucial, role alongside marketing efforts that could shape public expectations and demand. In our review, we also found that financial incentives as a market-based approach that involves using economic motivations to influence behaviors were useful (52). Similarly, interventions that altered incentives and enhanced accountability for providers were more effective in changing provider behaviors.

We found that a significant factor consistently influencing the delivery of healthcare services by IHCPs was the combination of interventions that mutually reinforced each other. For example, studies combined training with organizational changes to establish referral systems, integrating strategies such as accreditation and the provision of educational materials and resources. This approach proved effective across various subject areas and settings, ranging from establishing emergency trauma systems to assisting medicine sellers in managing sexually transmitted diseases to collaborating with traditional medical practitioners on family planning services (5, 18, 19). The range of studies on interventions with IHCPs is limited, indicating insufficient data to advocate for any specific intervention. Moreover, very few studies report on the costs of interventions, despite the necessity of this data to justify public funding. The overall impression is that the long-term effects of interventions with IHCPs are uncertain, and many of the strategies would be challenging to replicate. Very few studies provided detailed information about the interventions, such as training curricula, contents of birthing kits, or frequency of supervision. However, we found evidence supporting the effectiveness of IHCPs in certain medical conditions, such as lay health volunteers in tuberculosis, fever, and malaria management, and trained birth attendants in neonatal healthcare.

The studies included in our review do not definitively identify the most impactful interventions; however, it is evident that initiatives aimed at enhancing basic health service coverage through IHCPs or improving the quality of care they provide should be accompanied by rigorous evaluation research, publication of findings, and dissemination of lessons learned to maximize benefits (20, 21). The certainty of evidence generated was very low for all outcomes. Some interventions did not yield favorable results, and while study design issues might have played a role, other non-educational factors (e.g., deployment of anti-malarial medicine sellers and village health motivators or negotiation sessions) could indirectly hinder the promotion of appropriate dispensing in private pharmacies and medicine shops.

The strength of our study is the inclusion of a fairly large number of interventional studies across various LMICs and the comprehensive summary of the findings. The included studies were published between 1992 and 2021. The decision to search for a 35-year period was made to ensure a comprehensive review of the literature, capturing the historical evolution and long-term trends of interventions targeting IHCPs. While social changes in recent years are significant, the inclusion of earlier studies allows for a broader understanding of how interventions adapted over time. This wide margin provides a more complete picture of the evidence, identifying consistent patterns and emerging strategies that can inform current and future practices, despite the potential bias from earlier data. However, there are some limitations to this study. Only studies published in the English language were included. Changes in the practice of IHCPs over time contextualizing the societal perspective were not captured. Country-or context-specific interventions were not analyzed separately. The included studies had heterogeneities in the study populations, interventions, and outcomes and this debarred us from performing a meta-analysis. All required information was not available from all studies. Further limitations include the absence of providing a direction towards generalizability, sustainability, feasibility, and scalability of the interventions used across various studies and the certainty of evidence generated was very low for all outcomes.

There is a clear demand for high-quality intervention research targeting IHCPs. Given the high level of positive provider behaviors and knowledge among traditional birth attendants, further research into sustainable interventions and population-level outcomes with these providers would be particularly valuable. To achieve the greatest benefit, interventions should target providers most frequently used by the target demographic or for the target disease, rather than implementing blanket strategies across a geographic region (17, 21). Efforts should be directed toward identifying effective approaches for collaborating with traditional healers and unqualified doctors, who are relied upon by many. Given the constraints in resources to train and retain allopathic practitioners, both public and private health financiers must acknowledge the extensive market access and population interaction available to IHCPs. They have the potential to be valuable allies in extending the right to health for all individuals (22).

The practice of IHCPs raises ethical concerns about the quality of healthcare, accountability, and patient rights (23). The findings of this systematic review highlight the importance of addressing these considerations in policy and practice to safeguard patient outcomes and uphold ethical principles in the delivery of healthcare services. IHCPs must have adequate training and competency to deliver safe and effective care, respecting patients’ autonomy and right to informed decision-making, and addressing potential conflicts of interest, such as profit motives that may influence treatment decisions. Moreover, there is a need to balance the benefits of engaging IHCPs, such as improved access to care and cultural relevance, with potential risks, such as misdiagnosis, inappropriate treatment, and delays in seeking care from qualified healthcare professionals. The local context and situation also need to be addressed; for example, IHCPs were found to be useful in certain healthcare delivery during the Coronavirus Disease 2019 pandemic in LMICs (24, 25). By promoting transparency; accountability; and collaboration between IHCPs, formal healthcare providers, and regulatory bodies, various stakeholders can work together to ensure that patient care is delivered ethically and responsibly in a quality manner, ultimately improving health outcomes for underserved populations. It is essential to address the balance between the profit motives of IHCPs and the health goals of the public system.

The findings of this research may provide practical implications for various stakeholders in the healthcare system in LMICs. It is practical to recognize the role of IHCPs as primary care providers and the trust and acceptance they receive from the community (1–3). Regulatory bodies can establish guidelines for IHCPs to ensure compliance with quality care standards for fostering a people-centered health system (26). Often initiatives are taken from different stakeholders to train and integrate IHCPs as a part of the formal healthcare system (27). The Indian Medical Association does not endorse this (28) as this is illegal as per section 15.2(b) of the Indian Medical Council Act, 1965 (29). Healthcare providers and professional associations can collaborate with IHCPs to enhance care delivery, while community organizations and non-government organizations can implement targeted interventions to improve IHCP capacity. Overall, our findings can inform policy, practice, and community engagement efforts, aiming to improve the delivery of healthcare services by IHCPs and address health disparities for underserved populations.

## 5 Conclusion

In many LMICs, IHCPs often serve as the initial point of care for common illnesses like diarrhea, fever, and cough in children, as well as for family planning and reproductive healthcare in adults. Several multifaceted interventions coupled with training showed improvements in the delivery of healthcare services by IHCPs. However, the improvements were inconsistent across studies and the certainty of evidence generated was very low for all outcomes. Based on the review, it is difficult to identify any context-specific optimum intervention strategy to improve the delivery of healthcare services by IHCPs in LMICs. Implementing additional strategies, such as increased regulatory oversight, or the establishment of referral systems to qualified providers, alongside training, could enhance the delivery of healthcare services by IHCPs.

## Data Availability

The original contributions presented in the study are included in the article/Supplementary material, further inquiries can be directed to the corresponding author.
