# Supplementary material for: Interventions on informal healthcare providers to improve the delivery of healthcare services in low-and middle-income countries: a systematic review
Source: Front Public Health. 2024 Oct 1;12:1456868. doi: 10.3389/fpubh.2024.1456868 (PMC11473302; doi:10.3389/fpubh.2024.1456868)
Supplement: Supplementary file 1 [file Data_Sheet_1.docx]

Supplementary Material

**Table s1.** Search terms used in the selected databases.

| Population | - Midwives - Midwife - Traditional Birth Attendant - Birth Attendant, Traditional - Birth Attendants, Traditional - Traditional Birth Attendants - Medicine Practitioner, Traditional - Medicine Practitioners, Traditional - Practitioner, Traditional Medicine - Practitioners, Traditional Medicine - Traditional Medicine Practitioner - Traditional Health Practitioners - Health Practitioner, Traditional - Health Practitioners, Traditional - Practitioner, Traditional Health - Practitioners, Traditional Health - Traditional Health Practitioner - Traditional Healers - Healer, Traditional - Healers, Traditional - Traditional Healer - African Witch Doctor - African Witch Doctors - Doctor, African Witch - Doctors, African Witch - Witch Doctors, African - Witch Doctor, African - Complementary and Alternative Medicine Practitioners - CAM Practitioners - CAM Practitioner - Practitioner, CAM - Practitioners, CAM - Ethnobotanists - Ethnobotanist - Complementary Health Practitioners - Complementary Health Practitioner - Health Practitioner, Complementary - Health Practitioners, Complementary - Practitioner, Complementary Health - Practitioners, Complementary Health - Shamans - Shaman - Phytotherapists - Phytotherapist - Indigenous Medicine Practitioners - Indigenous Medicine Practitioner - Medicine Practitioner, Indigenous - Medicine Practitioners, Indigenous - Practitioner, Indigenous Medicine - Practitioners, Indigenous Medicine - Alternative Medicine Practitioners - Alternative Medicine Practitioner - Medicine Practitioner, Alternative - Medicine Practitioners, Alternative - Practitioner, Alternative Medicine - Practitioners, Alternative Medicine - Complementary Medicine Practitioners - Complementary Medicine Practitioner - Medicine Practitioner, Complementary - Medicine Practitioners, Complementary - Practitioner, Complementary Medicine - Practitioners, Complementary Medicine - Medicine Men - Medicine Mens - Men, Medicine - Mens, Medicine - Herbalists - Herbalist - Pharmacist - Clinical Pharmacists - Clinical Pharmacist - Pharmacist, Clinical - Pharmacists, Clinical - Community Pharmacists - Community Pharmacist - Pharmacist, Community - Pharmacists, Community - Retail Pharmacists - Pharmacist, Retail - Pharmacists, Retail - Retail Pharmacist - Care, Patient - Informal care - Informal cares - care, Informal - cares, Informal - Community Health Worker - Health Worker, Community - Health Workers, Community - Worker, Community Health - Workers, Community Health - Community Health Aides - Aide, Community Health - Aides, Community Health - Community Health Aide - Health Aide, Community - Health Aides, Community - Family Planning Personnel - Personnel, Family Planning - Planning Personnel, Family - Village Health Workers - Health Worker, Village - Health Workers, Village - Worker, Village Health - Workers, Village Health - Village Health Worker - Barefoot Doctors - Barefoot Doctor - Doctor, Barefoot - Doctors, Barefoot - Family Planning Personnel Characteristics |
| --- | --- |
| Intervention | - Workshops - Workshop - Training Programs - Program, Training - Programs, Training - Training Program - Educational Activities - Activities, Educational - Activity, Educational - Educational Activity - Literacy Programs - Literacy Program - Program, Literacy - Programs, Literacy |

**Table s2.** Risk of bias of the quasi-experimental studies as per the ROBINS-I tool.

| **Studies** | **Bias due to confounding** | **Bias in selection of participants into the study** | **Bias in classification of interventions** | **Bias due to deviations from intended interventions** | **Bias due to missing data** | **Bias in measurement of outcomes** | **Bias in selection of the reported result** | **Overall bias** |
| --- | --- | --- | --- | --- | --- | --- | --- | --- |
| Ratanajamit, 1972 | Low | Moderate | Moderate | Low | Low | Low | Low | Moderate |
| Oshiname, 1992 | Low | Moderate | Moderate | Low | Low | Serious | Low | Serious |
| Podhipak, 1993 | Low | No information | Moderate | Low | Low | Low | Low | Moderate |
| Kambo, 1994 | Moderate | Moderate | Moderate | Serious | Low | Low | Low | Serious |
| Lynch, 1994 | Low | Moderate | Moderate | Low | Low | Serious | Low | Serious |
| Alisjahbana 1995 | Low | No information | Moderate | Low | Low | Low | Low | Moderate |
| Kumar, 1995 | Moderate | Moderate | Moderate | Low | Low | Low | Low | Moderate |
| Matthews, 1995 | Low | Moderate | Moderate | Low | Low | Serious | Low | Serious |
| Miller, 1995 | Low | Low | Low | Low | Low | Low | Low | Low |
| Pick, 1996 | Moderate | Moderate | Moderate | Low | Low | Low | Low | Moderate |
| Nations, 1997 | Moderate | Moderate | Moderate | Low | Low | Moderate | Moderate | Moderate |
| Somse, 1998 | No information | No information | Moderate | Low | Low | Low | Low | Moderate |
| Marsh, 1999 | Moderate | Moderate | Moderate | Low | Low | Moderate | Moderate | Moderate |
| Schaider, 1999 | Low | Serious | Moderate | Low | Low | Serious | Low | Serious |
| Smith, 2000 | Low | Low | Low | Low | Low | Low | Low | Low |
| Bailey, 2002 | Moderate | Moderate | Moderate | Low | Low | Moderate | Moderate | Moderate |
| Kaona, 2003 | Low | Serious | Moderate | Low | Low | Serious | Low | Serious |
| Tavrow, 2003 | No information | No information | Moderate | Low | Low | Low | Low | Moderate |
| Tumwikirize, 2004 | Low | No information | Moderate | No information | Low | Low | Low | Moderate |
| Peltzer, 2006 | Low | Moderate | Moderate | Serious | Low | Serious | Low | Serious |
| Salim, 2006 | Low | Moderate | Moderate | Low | Low | Serious | Low | Serious |
| Tawfik, 2006 | Low | Low | Low | Low | Low | Low | Low | Low |
| Mbonye, 2007 | Low | Low | No information | Low | Low | Low | Moderate | Moderate |
| Nsimba, 2007 | Low | No information | Moderate | Low | Low | Low | No information | Moderate |
| Onwujekwe, 2007 | Low | Low | Moderate | Low | Moderate | Low | Low | Moderate |
| Sima, 2019 | Low | Low | Low | Low | Low | Low | Low | Low |

**Table s3.** Risk of bias of the randomized controlled trials as per the Cochrane risk of bias tool 2.

| **Author, year** | **Bias arising from the randomization process** | **Bias due to deviations from intended interventions** | **Bias due to missing outcome data** | **Bias due to measurement of the outcome** | **Bias in selection of the reported result** | **Overall bias** |
| --- | --- | --- | --- | --- | --- | --- |
| Adu-Sarkodie, 2000 |  |  |  |  |  |  |
| Singhal, 2001 |  |  |  |  |  |  |
| Chalker, 2002 |  |  |  |  |  |  |
| Garcia, 2003 |  |  |  |  |  |  |
| Poudyal, 2003 |  |  |  |  |  |  |
| Chalker, 2005 |  |  |  |  |  |  |
| Jokhio, 2005 |  |  |  |  |  |  |
| Shah, 2007 |  |  |  |  |  |  |
| Abuya, 2009 |  |  |  |  |  |  |
| Das, et al., 2016 |  |  |  |  |  |  |
| Talukder, 2017 |  |  |  |  |  |  |
| Sundararajan, 2021 |  |  |  |  |  |  |

| **Index** | |
| --- | --- |
|  | Low risk of bias |
|  | Moderate risk of bias |
|  | High risk of bias |

**Table s4.** Certainty of the evidence for changes in knowledge, attitude, and practice of appropriate case diagnosis and management generated as per the GRADE (Grading of Recommendations Assessment, Development and Evaluation) approach.

| **GRADE domain** | **Judgement** | **Concerns about certainty domains** |
| --- | --- | --- |
| Methodological limitations of the studies | One out of 28 studies, [five](https://www.ncbi.nlm.nih.gov/pmc/articles/PMC5502230/#R7) had a serious risk of bias. | Serious |
| Indirectness | The population, intervention, and comparator in all studies provide direct evidence to the clinical question at hand. However, the population was heterogeneous. The intervention was educational program or training or outreach program. The outcome assessment was measured in different ways across the studies. | Not serious, borderline |
| Imprecision | Some studies reported improvements, while some did not. The sample size varied widely. | Serious |
| Inconsistency | The direction and magnitude of the effect of intervention varied across different studies. | Serious |
| Publication bias | This was not suspected because both negative and positive studies were published, and the search strategy was robust. | Not suspected |

**Table s5.** Certainty of the evidence for improved referral services generated as per the GRADE (Grading of Recommendations Assessment, Development and Evaluation) approach.

| **GRADE domain** | **Judgement** | **Concerns about certainty domains** |
| --- | --- | --- |
| Methodological limitations of the studies | One out of seven studies, [one](https://www.ncbi.nlm.nih.gov/pmc/articles/PMC5502230/#R7) had a serious risk of bias. | Serious |
| Indirectness | The population, intervention, and comparator in all studies provide direct evidence to the clinical question at hand. However, the population was heterogeneous. The intervention was educational program or training or outreach program. The outcome assessment was measured in different ways across the studies. | Not serious, borderline |
| Imprecision | Some studies reported improvements, while some did not. The sample size varied widely. | Serious |
| Inconsistency | The direction and magnitude of the effect of intervention varied across different studies. | Serious |
| Publication bias | This was not suspected because both negative and positive studies were published, and the search strategy was robust. | Not suspected |

**Table s6.** Certainty of the evidence for effective contraceptive use generated as per the GRADE (Grading of Recommendations Assessment, Development and Evaluation) approach.

| **GRADE domain** | **Judgement** | **Concerns about certainty domains** |
| --- | --- | --- |
| Methodological limitations of the studies | One out of seven studies, [one](https://www.ncbi.nlm.nih.gov/pmc/articles/PMC5502230/#R7) had a serious risk of bias. | Serious |
| Indirectness | The population, intervention, and comparator in all studies provide direct evidence to the clinical question at hand. However, the population was heterogeneous. The intervention was educational program or training or outreach program. The outcome assessment was measured in different ways across the studies. | Not serious, borderline |
| Imprecision | Some studies reported improvements, while some did not. The sample size varied widely. | Serious |
| Inconsistency | The direction and magnitude of the effect of intervention varied across different studies. | Serious |
| Publication bias | This was not suspected because both negative and positive studies were published, and the search strategy was robust. | Not suspected |

**Table s7.** Certainty of the evidence for medication appropriateness generated as per the GRADE (Grading of Recommendations Assessment, Development and Evaluation) approach.

| **GRADE domain** | **Judgement** | **Concerns about certainty domains** |
| --- | --- | --- |
| Methodological limitations of the studies | One out of eight studies, [one](https://www.ncbi.nlm.nih.gov/pmc/articles/PMC5502230/#R7) had a serious risk of bias. | Serious |
| Indirectness | The population, intervention, and comparator in all studies provide direct evidence to the clinical question at hand. However, the population was heterogeneous. The intervention was educational program or training or outreach program. The outcome assessment was measured in different ways across the studies. | Not serious, borderline |
| Imprecision | Some studies reported improvements, while some did not. The sample size varied widely. | Serious |
| Inconsistency | The direction and magnitude of the effect of intervention varied across different studies. | Serious |
| Publication bias | This was not suspected because both negative and positive studies were published, and the search strategy was robust. | Not suspected |

**Table s8.** Summary of findings for all outcomes according to the GRADE (Grading of Recommendations Assessment, Development and Evaluation) approach.

| **Outcome** | **Effect** | **Number of studies** | **Certainty in the evidence^*^** |
| --- | --- | --- | --- |
| Changes in knowledge, attitude, and practice of appropriate case diagnosis and management | There were heterogeneities in the population, intervention, and outcome assessment. The studies showed inconsistent effect | 28 | VERY LOW ⊕OOO (due to serious risk of methodological limitations, imprecision, and inconsistency) |
| Improved referral services | There were heterogeneities in the population, intervention, and outcome assessment. The studies showed inconsistent effect | 7 | VERY LOW ⊕OOO (due to serious risk of methodological limitations, imprecision, and inconsistency) |
| Effective contraceptive use | There were heterogeneities in the population, intervention, and outcome assessment. The studies showed inconsistent effect | 7 | VERY LOW ⊕OOO (due to serious risk of methodological limitations, imprecision, and inconsistency) |
| Medication appropriateness | There were heterogeneities in the population, intervention, and outcome assessment. The studies showed inconsistent effect | 8 | VERY LOW ⊕OOO (due to serious risk of methodological limitations, imprecision, and inconsistency) |

**Table s9.** Details of the interventions and outcomes based on the World Health Organization (WHO) Health Intervention Classification Framework categories.

| **Education and Training: activities aimed at improving knowledge and skills** |
| --- |
| - Oshiname 1992 (Nigeria): Training for Patent Medicine Vendors aimed at improving their knowledge of treatment for malaria, diarrhea, STDs, respiratory infections, and malnutrition, including prescription reading and medication counseling. - Podhipak 1993 (Thailand): Intervention program for Pharmacists and Medicine Sellers focusing on changes in prescribing practices, particularly for ORS, antibiotics, and antidiarrheal medicines. - Kambo 1994 (India): Training for Traditional Medical Practitioners to enhance their knowledge of contraceptive use. - Lynch 1994 (Uganda): Training Traditional Birth Attendants (TBAs) to improve their performance and utilization, though with mixed results. - Alisjahbana 1995 (Indonesia): Comprehensive training for informal care providers across all levels of the health care system, focusing on antenatal care, case referrals, and postnatal care improvements. - Kumar 1995 (India): Regular training sessions for TBAs focusing on case management of birth asphyxia and perinatal mortality. - Matthews 1995 (Nigeria): Training TBAs to improve identification of high-risk pregnancies and care for mothers and babies. - Miller 1995 (Pakistan): Training TBAs on perinatal outcomes, maternal nutrition, immunization, and hygiene. - Pick 1996 (Mexico): Intensive training for Pharmacists on HIV/AIDS knowledge retention and promoting condom sales. - Nations 1997 (Brazil): Multidisciplinary training for Afro-Brazilian Umbanda Healers on AIDS prevention, risky behavior, and alternative rituals. - Somse 1998 (Central African Republic): Training for Traditional Healers focusing on the treatment and prevention of AIDS and STDs. - Marsh 1999 (Kenya): Training shopkeepers on the appropriate sale of antimalarial and antipyretic medicines. - Schaider 1999 (Angola): Training TBAs on prenatal, delivery, and postnatal care, leading to a reduction in maternal mortality. - Adu-Sarkodie 2000 (Ghana): Training Pharmacists on the syndromic management of STIs. - Smith 2000 (Ghana): Training TBAs to improve maternal and perinatal outcomes. - Singhal 2001 (Philippines): Educational program for TBAs focusing on maternal care and neonatal practices. - Bailey 2002 (Guatemala): Training TBAs to enhance detection of obstetric complications and referrals. - Chalker 2002 (Vietnam): Multi-component training for pharmacy staff on ARI, STD, and antibiotic management. - Ratanajamit 2002 (Thailand): Educational program for medicine store personnel on emergency contraception. - Garcia 2003 (Peru): Training pharmacy workers on STD recognition and management. - Kaona 2003 (Zambia): Deputation and training of vendors and health motivators on malaria identification and chloroquine use. - Poudyal 2003 (Nepal): Training traditional healers to improve referral practices and knowledge of common illnesses, including HIV/AIDS. - Tavrow 2003 (Kenya): Outreach education for medicine wholesalers to improve malaria guideline compliance. - Tumwikirize 2004 (Uganda): Face-to-face educational intervention for medicine counter attendants on ARI management in children. - Chalker 2005 (Vietnam, Thailand): Multi-faceted training for private pharmacy sellers focusing on illegal steroid and antibiotic dispensing. - Jokhio 2005 (Pakistan): Training TBAs to reduce perinatal and maternal mortality through better identification of danger signs in pregnancy. - Peltzer 2006 (South Africa): Training traditional healers on HIV/AIDS, STI, and TB prevention strategies. - Salim 2006 (Bangladesh): Training village doctors to improve TB referral and treatment quality. - Tawfik 2006 (Uganda): Negotiation sessions for private practitioners to improve management of childhood diarrhea, ARI, and malaria. - Mbonye 2007 (Uganda): Community-based training for TBAs, medicine shop vendors, and reproductive health workers on malaria prevention during pregnancy. - Nsimba 2007 (Tanzania): Educational intervention for medicine sellers to improve adherence to malaria and childhood illness treatment guidelines. - Onwujekwe 2007 (Kenya): Training community health workers to provide low-cost malaria treatment. - Shah 2007 (Pakistan): Training non-formal care providers in syndromic management of STDs. - Abuya 2009 (Kenya): Training medicine retailers on antimalarial sales and public information campaigns. - Das et al. 2016 (India): Multitopic training program for informal providers focusing on adherence to checklists, correct case management, and reducing unnecessary medicine use. - Talukder 2017 (Bangladesh): Training and supervision for TBAs and community volunteers to improve breastfeeding practices. - Sima 2019 (Ethiopia): Training traditional healers to improve TB case detection and referral. - Sundararajan 2021 (Uganda): Educational training for traditional healers on HIV point-of-care testing. |
| **Health Services: direct health care services provided to individuals** |
| - Sundararajan 2021 (Uganda): Post-training delivery of point-of-care HIV tests by traditional healers, significantly increasing the number of tests administered. |
| **Policy and Guidelines: Implementation of policies or guidelines to improve health outcomes** |
| - Abuya 2009 (Kenya): Implementation of training workshops and public information campaigns on malaria treatment, leading to improved sales practices and knowledge among medicine retailers. |
| **Community-based Interventions: Activities that engage community members in health promotion and disease prevention** |
| - Mbonye 2007 (Uganda): Implementation of a community-based delivery system for preventive treatment of malaria in pregnancy, improving treatment adherence and antenatal care coverage. |
